# *Litopenaeus vannamei* Sterile-Alpha and Armadillo Motif Containing Protein (LvSARM) Is Involved in Regulation of *Penaeidins* and *antilipopolysaccharide factors*

**DOI:** 10.1371/journal.pone.0052088

**Published:** 2013-02-06

**Authors:** Pei-Hui Wang, Zhi-Hua Gu, Ding-Hui Wan, Wei-Bin Zhu, Wei Qiu, Shao-Ping Weng, Xiao-Qiang Yu, Jian-Guo He

**Affiliations:** 1 MOE Key Laboratory of Aquatic Product Safety/State Key Laboratory of Biocontrol, School of Life Sciences, Guangzhou, People’s Republic of China; 2 School of Marine Sciences, Sun Yat-Sen University, Guangzhou, People’s Republic of China; 3 Division of Cell Biology and Biophysics, School of Biological Sciences, University of Missouri-Kansas City, Kansas City, Missouri, United States of America; Centro de Investigacion y de Estudios Avanzados del Instituto Politecnico Nacional, Mexico

## Abstract

The Toll-like receptor (TLR)-mediated NF-κB pathway is tightly controlled because overactivation may result in severe damage to the host, such as in the case of chronic inflammatory diseases and cancer. In mammals, sterile-alpha and armadillo motif-containing protein (SARM) plays an important role in negatively regulating this pathway. While *Caenorhabditis elegans* SARM is crucial for an efficient immune response against bacterial and fungal infections, it is still unknown whether *Drosophila* SARM participates in immune responses. Here, *Litopenaeus vannamei* SARM (LvSARM) was cloned and functionally characterized. LvSARM shared signature domains with and exhibited significant similarities to mammalian SARM. Real-time quantitative PCR analysis indicated that the expression of *LvSARM* was responsive to *Vibrio alginolyticus* and white spot syndrome virus (WSSV) infections in the hemocyte, gill, hepatopancreas and intestine. In *Drosophila* S2 cells, LvSARM was widely distributed in the cytoplasm and could significantly inhibit the promoters of the NF-κB pathway-controlled antimicrobial peptide genes (AMPs). Silencing of *LvSARM* using dsRNA-mediated RNA interference increased the expression levels of *Penaeidins* and *antilipopolysaccharide factors*, which are *L.vannamei* AMPs, and increased the mortality rate after *V. alginolyticus* infection. Taken together, our results reveal that LvSARM may be a novel component of the shrimp Toll pathway that negatively regulates shrimp AMPs, particularly *Penaeidins* and *antilipopolysaccharide factors*.

## Introduction

Innate immunity is the body’s first line of defense against pathogens [1], [2]. This immune response relies on germ line-encoded pattern recognition receptors (PRRs), such as the Toll-like receptors (TLRs), which participate in the recognition of pathogen-associated molecular patterns (PAMPs) [1], [2]. In mammals, various PAMPs derived from viruses, bacteria, fungi and protozoa can be detected by distinct TLRs, leading to the activation of NF-κB [3]. This transcription factor has a central role in coordinating the expression of proinflammatory cytokines and chemokines to eliminate microbial infection by provoking inflammation and recruiting innate and adaptive immune cells [3], [4].

In *Drosophila*, Gram-positive bacteria, fungi and certain viruses can activate the Toll pathway [5], [6]. The recognition of Gram-positive bacteria and fungi by peptidoglycan recognition proteins (PGRPs) and Gram-negative bacteria-binding proteins (GNBPs), but not by Toll itself, triggers a proteolytic cascade that cleaves the endogenous Toll ligand Spätzle. This ligand binds to the Toll receptor, leading to activation of the NF-κB family protein Dorsal [5], [6]. Activated Dorsal then translocates to the nucleus to promote the expression of immune-related genes, such as those encoding antimicrobial peptide genes (AMPs) [6]. Although no component of the *Drosophila* Toll pathway has been identified for the detection of viruses, certain viruses can also activate the Toll-Dorsal pathway and induce AMP expression [5], [7]–[9].

Surprisingly, the classical Toll pathway does not seem to be conserved in *Caenorhabditis elegans* [10]–[12]. More specifically, the *C. elegans* genome does not encode homologs of the intracellular TLR adaptor protein myeloid differentiation primary response protein 88 (MyD88) or NF-κB-like transcription factors [10]. Meanwhile, Tol-1, the only Toll homolog in *C. elegans*, seems to play a major role in development but not an essential role in the control of immune responses [10]–[12].

Signaling downstream of mammalian TLRs, which leads to NF-κB activation, requires intracellular TLR adaptor proteins with a Toll/interleukin-1 receptor (TIR) domain, including MyD88, TIR domain-containing adaptor inducing interferon-β (TRIF), MyD88-adaptor-like (MAL), TRIF-related adaptor molecule (TRAM) and sterile-alpha and armadillo motif containing protein (SARM) [4], [13], [14]. MyD88 is a universal adaptor protein in the downstream signaling of various TLRs, with the exception of TLR3, which instead recruits TRIF [3], [4], [13]. TLR2 and TLR4 signaling requires MAL, which bridges TLR and MyD88 [3], [4], [13]. In addition to triggering MyD88-dependent signaling, TLR4 elicits TRIF-dependent signaling. In the latter signaling pathway, TLR4 needs TRAM to activate TRIF. Both MyD88- and TRIF-dependent signaling lead to NF-κB activation [3], [4], [13].

SARM is the only negative regulator of the five TLR adaptor proteins, functioning as an inhibitor of TRIF-dependent signaling by associating with TRIF [13]–[16]. As a result, knockdown of endogenous SARM leads to NF-κB activation and enhanced cytokine and chemokine induction [15]. Among the five TLR adaptor proteins, SARM is the only TIR domain-containing protein conserved from *C. elegans* to mammals [10], [17], [18]. Knockdown of the *C. elegans* SARM homolog *TIR-1* results in decreased *C. elegans* survival during fungal and bacterial infections, which has been related to reduced expression of two AMPs, NLP-29 and NLP-31 [10], [18], [19]. However, this effect is independent of the *C. elegans* Toll homolog Tol-1 but dependent on the p38 mitogen-activated protein kinase (MAPK) pathway, which regulates innate immune responses in evolutionarily diverse species [10], [18], [19].

*Drosophila* expresses the two TIR domain-containing adaptor proteins MyD88 and SARM. Similar to mammalian MyD88, *Drosophila* MyD88 can activate the classical Toll pathway and induce AMP expression in response to fungal and Gram-positive bacterial infections [5], [20]. However, whether *Drosophila* SARM participates in the classical Toll pathway is still unknown [18]. In another arthropod, *Carcinoscorpius rotundicauda*, a SARM ortholog was reported to suppress NF-κB activation through downregulation of the TRIF-dependent TLR signaling pathway after expression in human cells [17].

The culture of penaeid shrimp is rapidly developing as a major business endeavor worldwide. However, various shrimp diseases, caused by white spot syndrome virus (WSSV), *Vibrio* spp., *Aeromonas* and others, have resulted in high mortality and huge economic losses [21], [22]. A better understanding of the immune responses induced by these microbes would aid in the design of better strategies for the prevention and control of shrimp diseases [22]–[24]. Penaeidins (PENs), a family of AMPs from penaeid shrimp, possess antifungal, antibacterial and antiviral activities and even show proinflammatory cytokine features by attracting shrimp granulocytes toward the inflammatory site and by promoting adhesion [25]–[29]. Antilipopolysaccharide factors (ALFs) belong to another family of AMPs and are the key effector molecules of the innate immune system in crustaceans; ALFs also show antifungal, antibacterial and antiviral activities [30]–[33]. A recent study suggests that ALFs may act as cytokine-like regulatory molecules and as effector molecules [33]. Some shrimp AMPs, including PENs and ALFs, are activated in response to microbial infections, and their expression levels are related to successful host immune responses [34], [35]. In the model crustacean *Litopenaeus vannamei*, several components of the Toll pathway have been reported, including LvToll1-3, Spätzles (LvSPZ1-3), the NF-κB family protein LvDorsal, tumor necrosis factor receptor-associated factor 6 (LvTRAF6) and IRAK family protein LvIRAK4 (or LvPelle) [36]–[40]. However, whether the shrimp Toll pathway is involved in the regulation of *PENs* and *ALFs*, similar to the regulation mechanism of *Drosophila AMPs* by the Toll pathway, is still elusive. Here, we performed cDNA cloning, expression analysis and functional studies of LvSARM from *L. vannamei*, demonstrating that LvSARM is a potential negative regulator of the Toll pathway in regulating the expression of shrimp *PENs* and *ALFs*.

## Materials and Methods

### 2.1. Animals

Healthy Pacific white shrimp (*L. vannamei*), each approximately 4–5 g in body weight, were purchased from a local shrimp farm in Zhuhai, Guangdong Province, China. The shrimp were cultured in a recirculating water tank system filled with air-pumped seawater (2.5% salinity) at 24–26°C and fed with commercial feed at 5% of body weight twice per day, as described previously [36], [37], [41]. The shrimp were cultured for at least seven days to allow them to acclimate before experiments were conducted.

### 2.2. RNA Extraction, cDNA Synthesis and Genomic DNA Extraction

*L. vannamei* total RNA was extracted from the gill using an RNeasy Mini Kit (Qiagen, Germany). Residual genomic DNA was removed using RNase-free DNase I (Qiagen, Germany). The cDNA template for rapid amplification of cDNA ends (RACE) PCR was prepared using a SMARTer™ RACE cDNA Amplification Kit (Clontech, USA). For gene cloning, the first strand cDNA was prepared using a PrimeScript™ 1st Strand cDNA Synthesis Kit (TaKaRa, China). For the real-time quantitative PCR (qPCR) analysis, the first strand cDNA was prepared using a PrimeScript™ RT Reagent Kit (TaKaRa, China). Genomic DNA was isolated from *L. vannamei* muscle using the Universal Genomic DNA Extraction Kit Ver. 3.0 (TaKaRa, China) according to the manufacturer’s instructions.

### 2.3. cDNA Cloning of LvSARM

In the NCBI expression sequence tag (EST) database for *L. vannamei*, three ESTs (Accession no. **FE053309, FE079166 and FE146806**) showing similarities to SARM were recovered. Based on the ESTs, we designed gene-specific primers (GSPs; listed in Table 1). The full-length cDNA of LvSARM was obtained using a 5′- and 3′-RACE approach, as performed in previous studies [36], [37], [41]. The genome sequence of LvSARM was obtained by a PCR application using the primers listed in Table 1.

**Table 1:** PCR primers used in this study.

Primer	Primer sequence (5′-3′)
**cDNA cloning**	
5′ LvSARM-RACE1	GTAAACAGCGGCGTTCCTC
5′ LvSARM-RACE2	GATGGGGATGCTGTCAACG
3′ LvSARM-RACE1	GTGGTCACCGTCAAGGAAGC
3′ LvSARM-RACE2	CCGTGAAGAACGAGGAGAACA
**Genome cloning**	
gLvSARMF1	GAGGAAGACTTTTCTCGTGCAT
gLvSARMR1	GCCACCATAGAGGGACAAGTT
gLvSARMF2	TGGAGGATGTTGTCAATGTCGC
gLvSARMR2	CGTGAGCCATTGGAACGT
gLvSARMF3	ATGCTGAATGCTGGCGTTAC
gLvSARMR3	TGGCCTGATTCAATTTTCTGT
**qPCR analysis**	
LvSARM-F	GTCCACATGCAGCTCAAAGA
LvSARM-R	ACTGGAGAGCTGCAACGATT
LvPEN2-F	GCATCAAGTTCGGAAGCTGT
LvPEN2-R	ACCCACATCCTTTCCACAAG
LvPEN3-F	CTCTGGCTTGTGGAATGGAT
LvPEN3-R	GCATGGATTCACTTCCTCGT
LvPEN4-F	ATGCTACGGAATTCCCTCCT
LvPEN4-R	ATCCTTGCAACGCATAGACC
LvALF1-F	ATAGTCGGGTTGTGGCACTC
LvALF1-R	GTCGTCCTCCGTGATGAGAT
LvALF2-F	CTGTGGAGGAACGAGGAGAC
LvALF2-R	CCACCGCTTAGCATCTTGTT
LvEF-1α-F	GAAGTAGCCGCCCTGGTTG
LvEF-1α-R	CGGTTAGCCTTGGGGTTGAG
**protein expression** *	
pAcLvSARM-F	CGGGGTACCCGCCACCATGGGGGTGGCAGGCGAG
pAcLvSARM-R	AAGGAAAAAAGCGGCCGCCAGTCCTTGGCAAAGTCTTTGTCGGA
**co-IP assays**	
pAcLvSARM-V5-F	CGGGGTACCCGCCACCATGGGGGTGGCAGGCGAG
pAcLvSARM-V5-R	AAGGAAAAAAGCGGCCGCCAGTCCTTGGCAAAGTCTTTGTCGGA
pAcLvSARM-Myc-F	CGGGGTACCCGCCACCATGGGGGTGGCAGGCGAG
pAcLvSARM-Myc-R	AAGGAAAAAAGCGGCCGCCATTACAGATCCTCTTCAGAGATGAGTTT-
	CTGCTCGTCCTTGGCAAAGTCTTTGTCGGA
pAcLvTRAF6-V5-F	GGGGTACCATGGAGAGTGTCGAAGAGTCCATTAC
pAcLvTRAF6-V5-R	GCTCTAGACACACAGCTTTGCTTCTCTATATG
pAcLvTRAF6-Myc-F	GGGGTACCATGGAGAGTGTCGAAGAGTCCATTAC
pAcLvTRAF6-Myc-R	GCTCTAGATTACAGATCCTCTTCAGAGATGAGTTTCTGCTCCACACA-
	GCTTTGCTTCTCTATATG

* Primers used in the cellular localization and the protein expression of luciferase reporter assays were the same for LvSARM.

### 2.4. Bioinformatics Analyses

Multiple sequence alignments were performed using the Clustal X 2.0 program (http://www.ebi.ac.uk/tools/clustalw2). The simple modular architecture research tool (SMART; http://smart.embl-heidelberg.de) was used to analyze the deduced amino acid sequences of LvSARM. Neighbor-joining (NJ) phylogenic trees were then constructed using MEGA 4.0 software (http://www.megasoftware.net/index.html). Bootstrap sampling was reiterated 1,000 times.

### 2.5. Real-time qPCR Analyses

Gram-negative *Vibrio alginolyticus* and WSSV inocula were prepared and quantified as in previous studies [36], [39]. In microbial challenge experiments, *L. vannamei* was injected intramuscularly at the third abdominal segment with 100 µl of *V. alginolyticus* inoculum (approximately 7×10^6^ CFU/shrimp) or with 100 µl of WSSV inoculum (approximately 10^7^ copies/shrimp). Untreated shrimp were used as controls. At 0, 3, 6, 12, 24, 36, 48 and 72 hours post-injection (hpi), five shrimp from each group were randomly selected for the gill, hemocyte, intestine, hepatopancreas and muscle collection. Healthy *L. vannamei* tissues, including the hemocyte, eyestalk, gill, heart, hepatopancreas, stomach, intestine, nerve, muscle, pyloric cecum and epithelium were collected for total RNA extraction for tissue distribution analysis of *LvSARM*. Total RNA isolation was performed, and PCR templates were prepared as described in Section 2.2. The expression of *LvSARM* was measured using the Master SYBR Green I system and a LightCycler (Roche), as in previous studies [36], [42]. Three replicate qPCRs were performed per sample, and three shrimp were analyzed per sample. The standard curves for *LvSARM* and *LvEF-1α* were generated by running triplicate reactions of a 10-fold dilution series (i.e., 10 different cDNA concentrations). The efficiencies for *LvSARM* and *LvEF-1α* were 1.924 and 2.023, respectively. The relative standard curve method was used to calculate fold changes in gene expression [42], [43].

### 2.6. Plasmid Construction

To express LvSARM in cells for cellular localization and functional studies, the pAc5.1-LvSARM vector was constructed using the pAc5.1/V5-His A vector (Invitrogen, USA) as described previously [41], [42]. We constructed an expression plasmid, pAc5.1-N-GFP, that could sufficiently express green fluorescent protein (GFP) in *Drosophila* S2 cells, as described in our previous study [36], [42]. The complete *LvSARM* ORF was inserted into the pAc5.1-N-GFP vector to create the pAc5.1-LvSARM-GFP vector, which expressed a fusion protein consisting of full-length LvSARM coupled to GFP. The complete ORF of *LvIMD*, an important component of the IMD pathway that functions in parallel to the Toll pathway to activate AMPs [41], was inserted into the pAc5.1-N-GFP vector to create the pAc5.1-LvIMD-GFP vector. Luciferase reporter vectors were constructed using PGL3-Basic vectors (Promega, USA), as described in our previous studies [36], [37], [41].

### 2.7. Cell Culture

Because no immortalized shrimp cell line is available at present, *Drosophila Schneider* 2 (S2) cells (Invitrogen), derived from a macrophage-like lineage, were used to analyze the cellular localization and function of LvSARM. *Drosophila* S2 cells were maintained at 28°C in Schneider’s *Drosophila* medium (SDM) (Invitrogen) without CO_2_ and supplemented with 10% fetal bovine serum (FBS). When the culture density reached approximately 6–20×10^6^ viable cells ml^−1^, the *Drosophila* S2 cells were passaged onto a new plate at a density of approximately 5×10^5^ viable cells ml^−1^.

### 2.8. Cellular Localization Analysis

*Drosophila* S2 cells were seeded onto poly-l-lysine-treated coverslips in 24-well plates 24 h before transfection. The cells were then transfected using Effectene Transfection Reagent (Qiagen, Germany) according to the manufacturer’s protocol. Specifically, plasmids pAc5.1-LvSARM-GFP and pAc5.1-LvIMD-GFP (control) were transfected into *Drosophila* S2 cells to investigate protein cellular localization. Thirty-six hours after transfection, the cells on the coverslips were washed twice with phosphate buffered saline (PBS), fixed in Immunol Staining Fix Solution (Beyotime, China) and stained with Hoechst 33258 (Beyotime, China). The coverslips were then examined for protein cellular localization using a Leica laser scanning confocal microscope.

### 2.9. Dual Luciferase Reporter Assays

*Drosophila* S2 cells were seeded onto a 96-well culture plate in 100 µl medium at 2×10^5^ cells ml^−1^ 24 h prior to transfection. To test whether LvSARM affects the promoters of NF-κB-controlled AMPs, the protein expression vector pAC5.1-LvSARM (0.05 µg per well) was cotransfected with the luciferase reporter gene pGL3-Basic, pGL3-PEN453, pGL3-PEN309, pGL3-PEN4, pGL3-Drs or pGL3-AttA (0.05 µg per well, respectively), all of which were constructed in previous studies and demonstrated to be predominantly regulated through NF-κB activation [36], [38], [41], [44]–[46]. The pRL-TK *Renilla* luciferase vector was used as an internal control. The cells were harvested and lysed 36 h after transfection to examine dual luciferase activities using the dual luciferase reporter assay system (Promega, USA).

### 2.10. Co-immunoprecipitation and Western Blot Analysis

*Drosophila* S2 cells cultured in 60 mm plates were transfected with various vector combinations (0.5 µg pAc5.1-LvSARM-Myc vector and 0.5 µg pAc5.1-LvTRAF6-V5 vector, 0.5 µg LvSARM-Myc vector and 0.5 µg pAc5.1 vector, 0.5 µg pAc5.1-LvTRAF6-Myc vector and 0.5 µg pAc5.1-LvSARM-V5 vector or 0.5 µg pAc5.1-LvTRAF6-Myc vector and 0.5 µg pAc5.1 vector). After 48 h, the cells were lysed, and co-immunoprecipitation (co-IP) experiments were performed using the ProFound™ c-Myc Tag IP/Co-IP Kit (Pierce, USA) according to the manufacturer’s instructions. Briefly, cell lysates and anti-c-Myc agarose were combined and incubated at 4°C overnight with constant inversion. On the following day, the samples were applied to centrifuge spin columns and washed three times with TBST. c-Myc-tagged proteins were eluted using non-reducing sample buffer. The elution products were then analyzed by sodium SDS-PAGE, followed by Western blotting using anti-V5 (1∶1,000) and anti-Myc (1∶1,000) monoclonal antibodies and a diaminobenzidine (DAB) substrate kit (Boster, China).

### 2.11. dsRNA Preparation and Silencing of LvSARM In Vivo by dsRNA-mediated RNAi

The double strand RNA (dsRNA) of *LvSARM* and *GFP* (dsLvSARM and dsGFP, respectively) were prepared using T7 RiboMAX Express (Promega) according to the manufacturer’s protocol. Briefly, DNA templates for the production of dsLvSARM and dsGFP were amplified by PCR using gene specific primers with the T7 RNA polymerase binding site at the 5′ terminus to produce sense and anti-sense RNA strands separately. The single-strand RNA was then annealed to generate dsRNA. After purification, the dsRNA was quantified and then stored at −80°C. For dsRNA-mediated RNA interference (RNAi) experiments, the experimental group (1–2 g per shrimp) was injected with dsLvSARM (1 µg/g shrimp) by intramuscular injection, while the control groups were injected with dsGFP and PBS, respectively. To determine the silencing effects, the gill samples from at least 3 shrimps of each treatment were collected at 0, 24, 72, 120 and 144 hours post-dsRNA injection (hpi), and total RNA was extracted. The total RNA from the gills of the dsRNA-injected *L. vannamei* was reverse-transcribed into the first strand cDNA for the expression analysis of *LvSARM*, *PENs* and *ALFs* using qPCR as described in Section 2.5.

### 2. 12. The Expression Level of L. vannamei PENs and ALFs in LvSARM Silenced Shrimp

The expression levels of *L. vannamei PENs* and *ALFs* (*LvPEN2*, *LvPEN3*, *LvPNE4, LvALF1* and *LvALF2*) were detected using the cDNA templates prepared in Section 2.11 by qPCR analysis. The primer amplification efficiencies for *LvPEN2*, *LvPEN3*, *LvPNE4, LvALF1* and *LvALF2* were 2.0, 1.981, 2.085, 1.997 and 1.953, respectively.

### 2. 13. The WSSV and V. alginolyticus Infection Experiments in dsRNA-injected L. vannamei

The efficiency of gene silencing in dsLvSARM-injected *L. vannamei* was significant compared with the control groups (>80%) at all the examined timepoints chosen for qPCR analysis. In the WSSV and *V. alginolyticus* infection experiments, we infected *L. vannamei* intramuscularly with a WSSV inoculum (approximately 10^7^ copies/shrimp) or *V. alginolyticus* (approximately 2×10^6^ CFU/shrimp) at 48 h after dsRNA injection and then recorded the mortality. Water exchange and feeding regimes were the same as described in Section 1.1.

### 2.14. Statistical Analyses

The data are presented as the mean ± standard error of the mean (SEM). A Student’s t-test was used to compare the means of two samples using Microsoft Excel. The chi-square statistic was performed to assess the differences in mortality rates by comparing the mortality of the dsLvSARM injection group with the PBS or dsGFP injection groups. In all cases, differences were considered to be significant when p<0.05 and highly significant when p<0.01.

## Results

### 3.1. Cloning and Sequence Analysis of LvSARM

**Figure 1:**
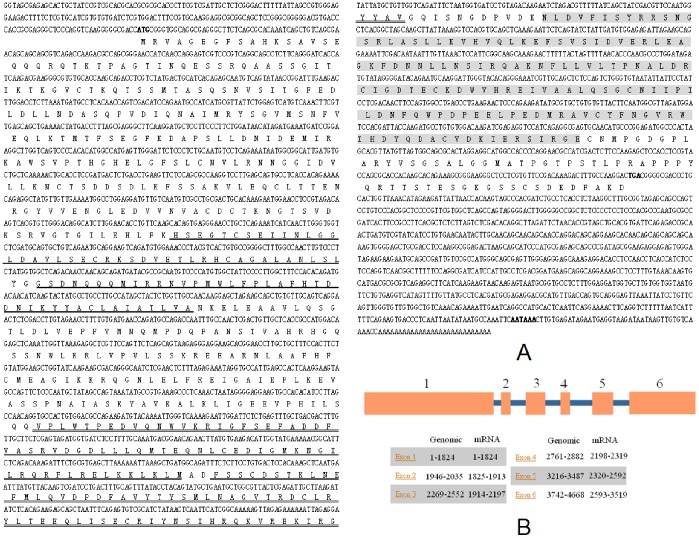
cDNA sequences (A) and genomic structure (B) of *LvSARM*. (A) The nucleotide (upper row) and deduced amino acid (lower row) sequences are shown. The initiation codon (ATG), stop codon (TGA) and the poly (A) signals (AATAAA) are shown in bold. Two ARM domains (residues 225–266 and 269–309, respectively), two SAM domains (residues 435–502 and 505–574, respectively) and the TIR domain (residues 586–727) of LvSARM are underlined, double underlined and shaded, respectively. (B) The genomic organization of *LvSARM*. The exons are depicted as boxes and introns as lines.

*LvSARM* full-length cDNA is 3519 bp long with an ORF of 2361 bp encoding a putative protein of 787 amino acids, a 5′-untranslated region of 199 bp, and a 3′-untranslated region of 959 bp (Fig. 1A). The sequence was deposited in the NCBI GenBank under accession no. **JN185615**. The *LvSARM* genome is 4668 bp, containing six exons and five introns (Fig. 1B). LvSARM shares 32.8%, 44.7%, 35.9%, and 38.0% identity with *C. elegans*, *C. rotundicauda*, *D. melanogaster*, and *Homo sapiens* SARM, respectively (Fig. S1). LvSARM contains two N-terminal ARM domains, two central SAM motifs, and a C-terminal TIR domain (Fig. S2). In comparison with CeSARM, CrSARM and DmSARM, LvSARM is shorter in length and thus more similar to HsSARM (Fig. S2).

### 3.2. Phylogenetic Tree Construction

**Figure 2:**
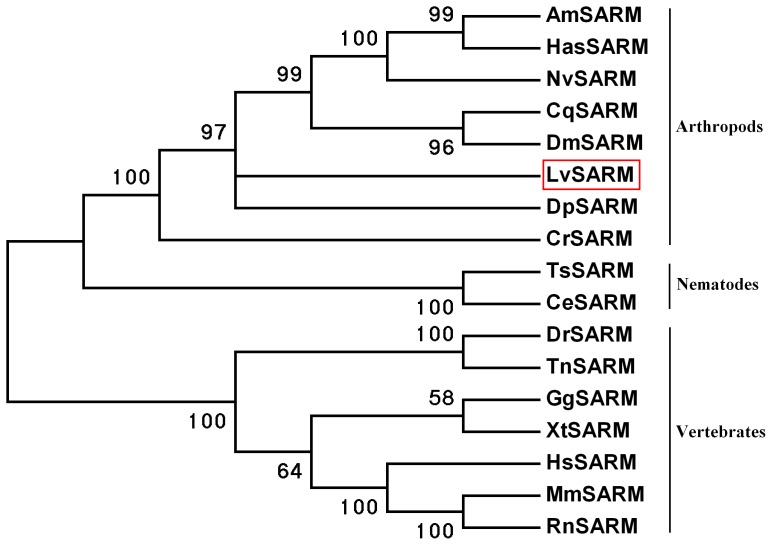
Phylogenetic tree analysis. LvSARM is indicated by a red box. CeSARM, *Caenorhabditis elegans* SARM (Accession no. **AAV91313**); DpSARM, *Daphnia pulex* SARM (Accession no. **EFX84452**); CrSARM, *Carcinoscorpius rotundicauda* SARM (Accession no. **ABB97045**); AmSARM, *Apis mellifera* SARM (Accession no. **XP_394430**); DmSARM, *Drosophila melanogaster* SARM (Accession no. **NM_001043129**); DrSARM, *Danio rerio* SARM (Accession no. **NP_001124068**); CgSARM, *Gallus gallus* SARM (Accession no. **XP_415814**); XtSARM, *Xenopus tropicalis* SARM (Accession no. **XM_002937143**); HsSARM, *Homo sapiens* SARM (Accession no. **NM_015077**).

Using MEGA 4.0 software, we constructed NJ phylogenetic trees for SARMs. The NJ phylogenetic tree for SARMs revealed that these proteins can be divided into three groups: arthropods, nematodes, and vertebrates. LvSARM was clustered with insect, *Daphnia pulex*, and *C. rotundicauda* SARM and belonged to the same arthropod group (Fig. 2).

### 3.3. Tissue Distribution of LvSARM in Healthy L. vannamei

**Figure 3:**
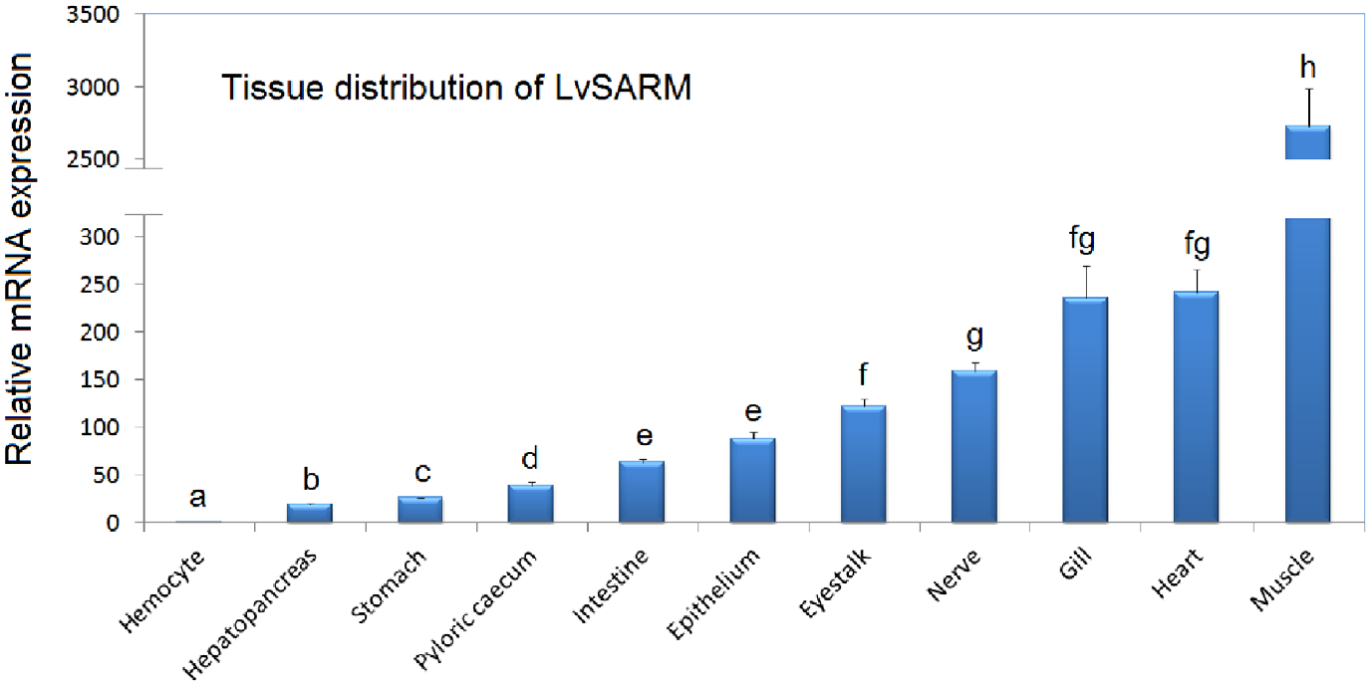
Tissue distribution of *LvSARM* in healthy *L. vannamei*. The expression of *LvSARM* in the hemocyte was set to 1.0. Bars with different letters indicate statistical differences.

In healthy shrimp, when normalized to mRNA expression in the hemocyte (1.0-fold), *LvSARM* was expressed at higher levels in the hepatopancreas (19.8-fold increase), stomach (26.0-fold), pyloric cecum (38.6-fold), intestine (63.2-fold), epithelium (87.7-fold), eyestalk (121.5-fold), nerve (159.4-fold), gill (235.9-fold), heart (241.0-fold) and muscle (2725.7-fold) (Fig. 3).

### 3.4. Expression Profiles of LvSARM after Microbial Challenges

**Figure 4:**
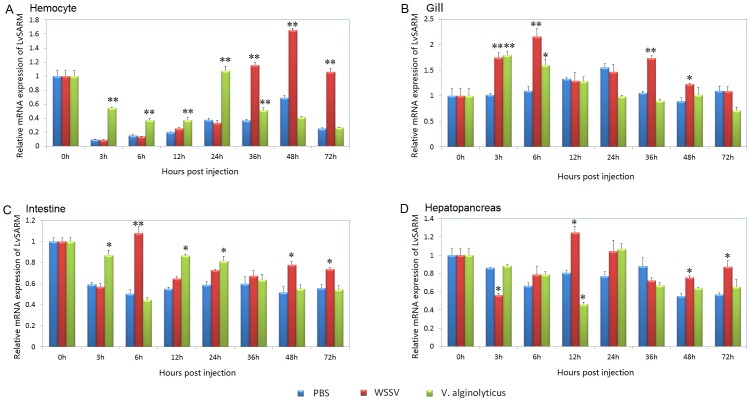
Expression of *LvSARM* in immune-challenged *L. vannamei*. Temporal expression of *LvSARM* in the hemocyte (A), gill (B), intestine (C) and hepatopancreas (D) after challenge with *V. alginolyticus* or WSSV. The expression of *LvSARM* in the samples injected with PBS, WSSV or *V. alginolyticus* at 0 hpi was set to 1.0 as the control. Expression values were normalized to those of *LvEF-1α* using the relative standard curve method. qPCR was performed in triplicate for each sample. The data are expressed as the mean fold change (means ± S.E., n = 3) relative to the untreated group. The statistical significance was calculated using Student’s t-test (*p<0.05; **p<0.01).5. Correlation between LvSARM Induction and Shrimp AMP Expression in the Hemocyte and Muscle.

In the hemocyte after PBS injection, the expression of *LvSARM* was greatly downregulated compared with the control group (Fig. 4A). After WSSV injection, *LvSARM* was downregulated at 3, 6, 12 and 24 hpi and recovered to normal levels at 36 and 72 hpi, but was upregulated at 48 hpi compared with healthy shrimp. Compared with the PBS injection group, *LvSARM* was upregulated 3.12-, 2.41- and 4.24-fold at 36, 48 and 72 hpi, respectively, after WSSV injection. After *V. alginolyticus* injection, *LvSARM* was downregulated at all timepoints examined compared to healthy shrimp, but upregulated 5.87-, 2.40-, 1.84-, 2.86- and 1.40-fold at 3, 6, 12, 24 and 36 hpi, respectively, compared to the PBS injection group. In the gill, after WSSV injection, *LvSARM* was upregulated at 3, 6 and 36 hpi compared with both the healthy shrimp and the PBS injection groups (Fig. 4B). After *V. alginolyticus* injection, *LvSARM* was also upregulated at 3 and 6 hpi compared with both the healthy shrimp and the PBS injection groups. In the hepatopancreas and intestine, the expression of *LvSARM* did not show obvious changes after PBS, WSSV or *V. alginolyticus* challenge (Fig. 4C and D).

**Figure 5:**
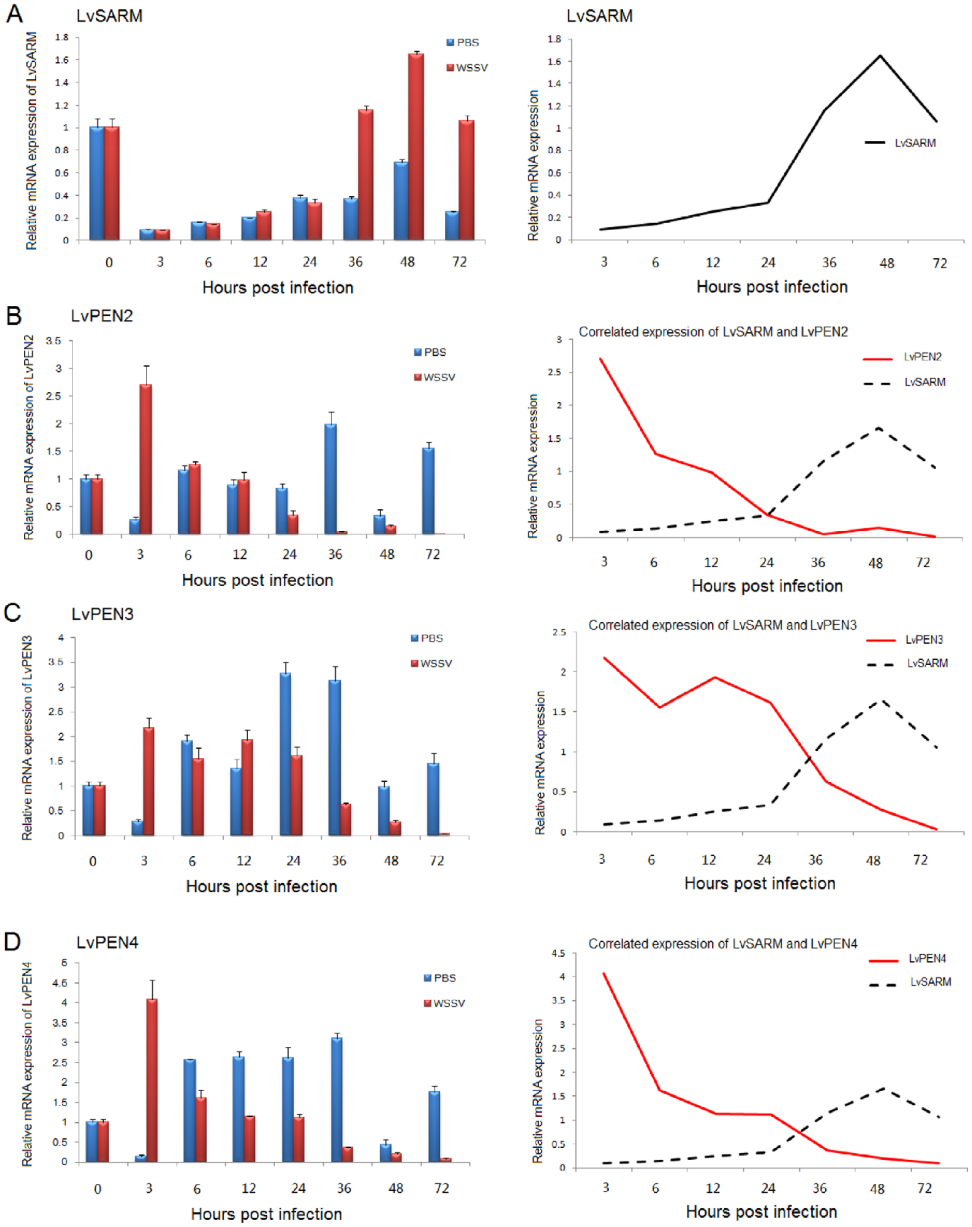
Expression of *LvSARM* (A), *LvPEN2* (B), *LvPEN3* (C) and *LvPEN4* (D) in the hemocyte after WSSV infection.

**Figure 6:**
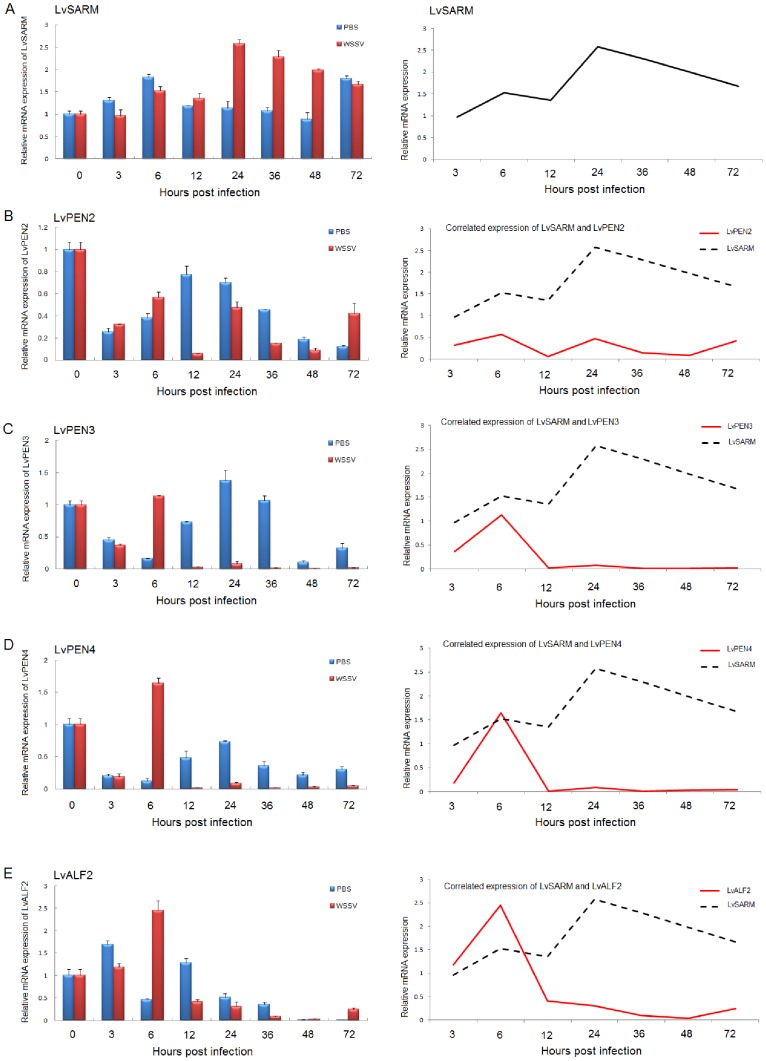
Expression of *LvSARM* (A), *LvPEN2* (B), *LvPEN3* (C), *LvPEN4* (D) and *LvALF2* (E) in the muscle after WSSV infection.

In the hemocyte, *LvSARM* was downregulated at 3 hpi, then its expression increased gradually from 6 to 72 hpi after WSSV infection (Fig. 5A). Meanwhile, we found shrimp AMPs including *LvPEN2*, *LvPEN3* and *LvPEN4* were downregulated from 6 to 72 hpi after WSSV infection (Fig. 5B, C, D). In the muscle, *LvSARM* was upregulated at 24, 36 and 48 hpi after WSSV infection (Fig. 6A). At the same time, we also observed that *LvPEN2*, *LvPEN3*, *LvPEN4* and *LvALF2* were downregulated (Fig. 6B, C, D, E).

### 3.6. Cellular Localization of LvSARM in Drosophila S2 Cells

**Figure 7:**
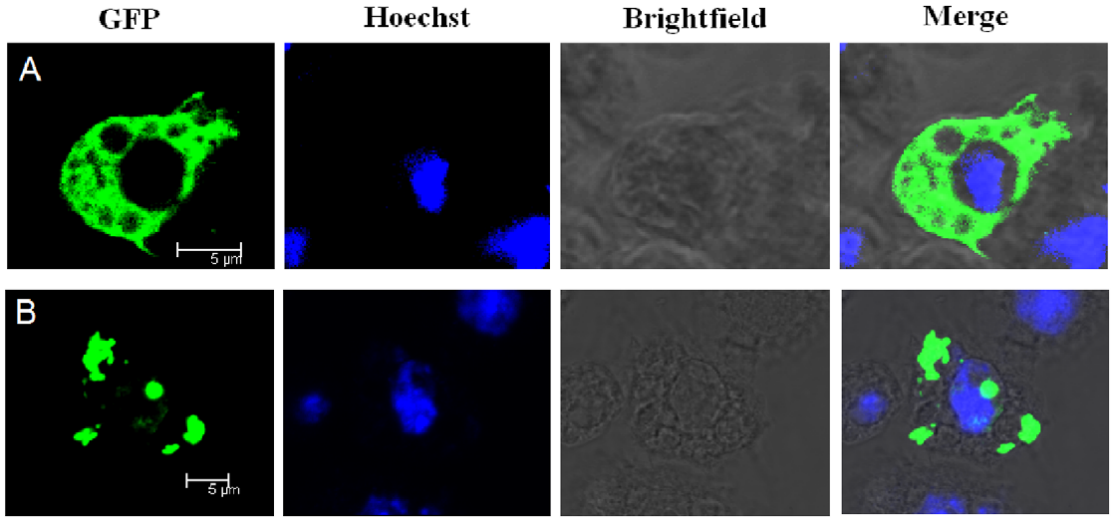
Subcellular localizations of LvSARM in *Drosophila* S2 cells. *Drosophila* S2 cells were transfected with plasmid pAc5.1-LvSARM-GFP. Nuclei were visualized with Hoechst (blue). (A) The LvSARM-GFP fusion protein was widely distributed in the cytoplasm of *Drosophila* S2 cells, as revealed by confocal microscopy. (B) *Drosophila* S2 cells transfected with pAc5.1-LvIMD-GFP (LvIMD Accession no. **ACL37048**) were used as controls.

Fluorescent imaging of the LvSARM-GFP fusion protein by confocal microscopy showed that LvSARM-GFP was ubiquitously distributed in the cytoplasm of *Drosophila* S2 cells (Fig. 7A). The positive control LvIMD-GFP fusion protein exhibited a cellular localization pattern different from that of LvSARM (Fig. 7B).

### 3.7. LvSARM Suppresses the Promoter Activities of Drosophila and Shrimp AMPs

**Figure 8:**
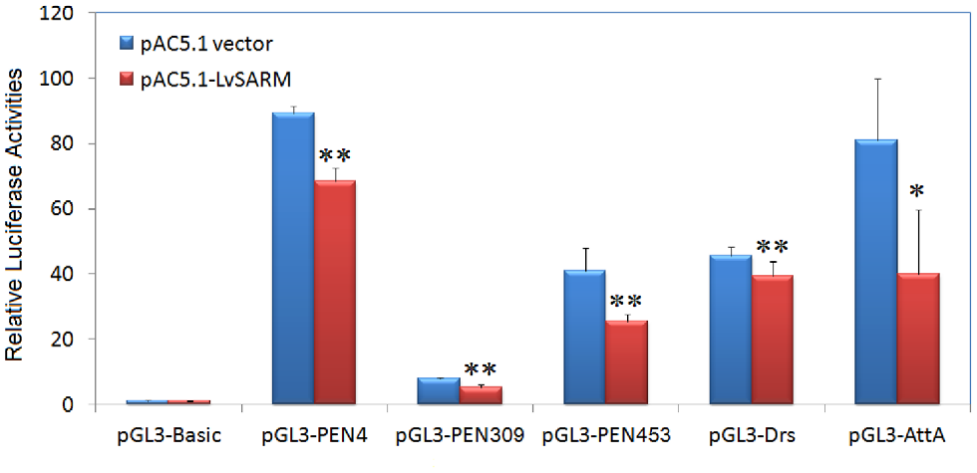
The effects of LvSARM on the promoter activities of *Drosophila* and shrimp AMPs in *Drosophila* S2 cells. *Drosophila* S2 cells were transfected with 0.05 µg of protein expression vector (pAC5.1 empty vector or pAC5.1-LvSARM vectors), 0.05 µg of reporter gene plasmid (pGL3-Basic, pGL3-PEN453, pGL3-PEN309, pGL3-PEN4 or pGL3-Drs or pGL3-AttA), and 0.005 µg of pRL-TK *Renilla* luciferase plasmid as an internal control (Promega, USA). Thirty-six hours later, the cells were harvested for examination of luciferase activities using the dual luciferase reporter assay system (Promega, USA). All data are representative of three independent experiments. The bars indicate the mean ± S.D. of the luciferase activity (n = 3). The statistical significance was calculated using Student’s t-test (*p<0.05; **p<0.01).

In *Drosophila* and shrimp, AMPs are important immune molecules, and their expression is believed to be mainly controlled by the Toll- and IMD-dependent NF-κB pathway. Here, we investigated whether LvSARM functions in signal transduction in the Toll-mediated NF-κB pathway in *Drosophila* S2 cells. Dual luciferase reporter assay results indicated that LvSARM inhibited the promoter activities of the *Drosophila* AMPs *Drosomycin* (*Drs*) and *Attacin A* (*AttA*), the *P. monodon* AMP *Penaeidin* (*PEN309* and *PEN453*), and the *L. vannamei* AMP *Penaeidin4* (*PEN4*) (Fig. 8).

### 3.8. LvSARM Associates with LvTRAF6

**Figure 9:**
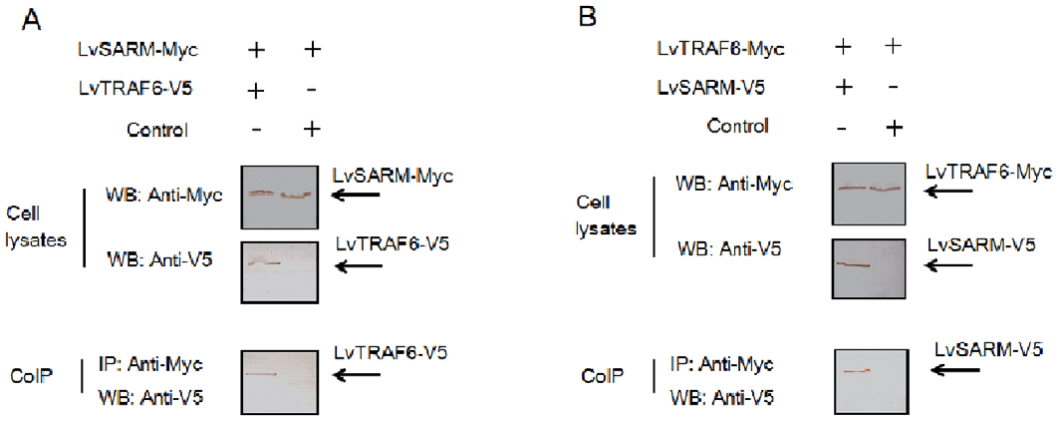
LvSARM associates with LvTRAF6. *Drosophila* S2 cells in a 60 mm plate were transfected with protein expression vectors. At 48 h post-transfection, the cells were lysed, and co-immunoprecipitation (co-IP) experiments were performed. Myc-tagged LvSARM co-precipitated with V5-tagged LvTRAF6 (A), and Myc-tagged LvTRAF6 co-precipitated with V5-tagged LvSARM (B). The pAc5.1 vectors were used as controls.

To investigate the molecular mechanism of LvSARM NF-κB suppression, we tested whether LvSARM associates with LvTRAF6 by co-IP. Myc-tagged LvSARM co-precipitated with V5-tagged LvTRAF6 (Fig. 9A), while Myc-tagged LvTRAF6 co-precipitated with V5-tagged LvSARM (Fig. 9B). We also observed that the pAC5.1 vector transfection groups did not show any co-precipitated proteins, serving as a negative control (Fig. 9). In conclusion, LvSARM might associate with LvTRAF6 in the Toll signaling pathway.

### 3.9. The Expression of LvSARM was Significantly Suppressed In Vivo by dsRNA-mediated RNAi

**Figure 10:**
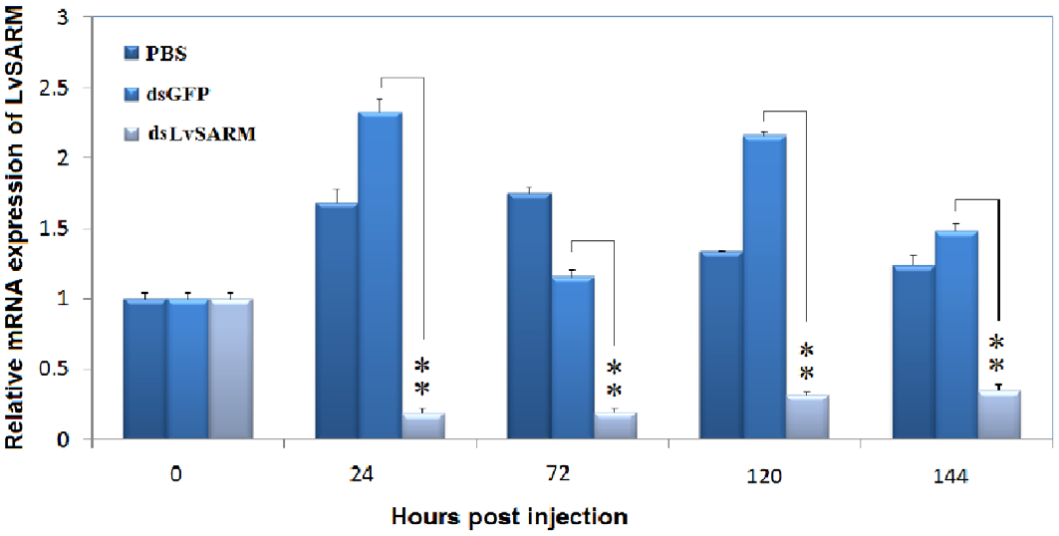
Expression of *LvSARM* in the gill of shrimp is significantly suppressed by dsRNA-mediated RNAi. At the indicated times after PBS, dsGFP (control) or dsLvSARM injection, total RNA was extracted from the gill and reverse transcribed to cDNA. The expression level of *LvSARM* was determined using qPCR. qPCR was performed in triplicate for each sample. The data are expressed as the mean fold change (means ± S.E., n = 3) relative to the dsGFP injection group. The statistical significance was calculated using Student’s t-test (*p<0.05; **p<0.01).

To further confirm the function of *LvSAM* in the regulation of shrimp AMPs PENs and ALFs, dsRNA-mediated RNAi experiments were performed. dsLvSARM (1 µg/g shrimp) was intramuscularly injected into shrimp in the experimental group, while injection of dsGFP or PBS was used in the control group. In the gill, the expression of *LvSARM* was significantly suppressed at 24, 72, 120 and 144 hpi (Fig. 10), while the expression of *LvSARM* was upregulated after dsGFP or PBS injection, which is consistent with the induced expression of *LvSARM* in the gill by PBS and WSSV (Fig. 4B).

### 3.10. Silencing of LvSARM Leads to Increased Expression of Shrimp AMPs PENs and ALFs in the Gill

**Figure 11:**
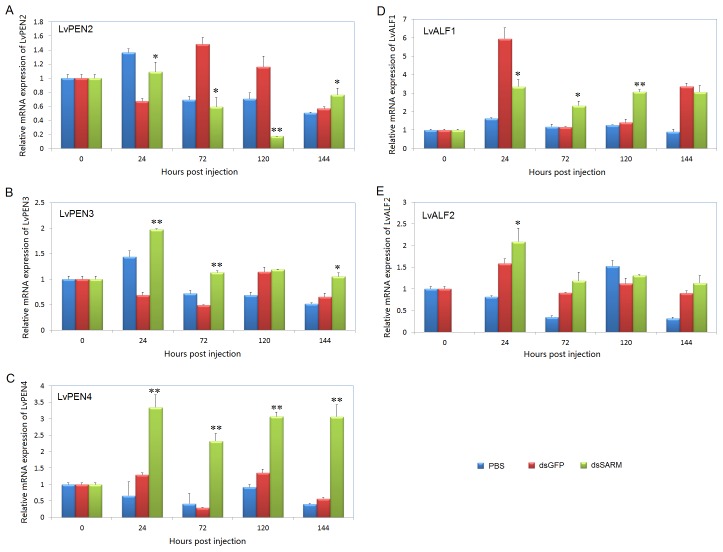
Silencing of *LvSARM* leads to increased expression of *LvPEN2*, *LvPEN3*, *LvPEN4*, *LvALF1* and *LvALF2* in the gill. The expression levels of *LvPEN2*, *LvPEN3*, *LvPEN4*, *LvALF1* and *LvALF2* were determined using qPCR. qPCR was performed in triplicate for each sample. The data are expressed as the mean fold change (means ± S.E., n = 3) relative to the dsGFP injection group. The statistical significance was calculated using Student’s t-test (*p<0.05; **p<0.01).

In the gill of dsRNA injected *L. vannamei*, the expression of *LvPEN2*, *LvPEN3*, *LvPEN4*, *LvALF1* and *LvALF2* was detected using qPCR. We observed that *LvPEN2* was significantly upregulated at 24 and 144 hpi but downregulated at 72 and 120 hpi compared with the dsGFP injection group (Fig. 11A). The expression of *LvPEN3* was upregulated 2.89-, 2.29-, 1.04- and 1.62-fold at 24, 72, 120 and 144 hpi, respectively, compared with the dsGFP injection group (Fig. 11B). The expression of *LvPEN4* was upregulated 2.59-, 8.01-, 2.26- and 5.40-fold at 24, 72, 120 and 144 hpi compared with the dsGFP injection group, respectively (Fig. 11C). The expression of *LvALF1* was upregulated 2.00- and 2.18-fold at 72 and 120 hpi compared with the dsGFP injection group, respectively (Fig. 11D). The expression of *LvALF2* was upregulated 1.32-, 1.31-, 1.66- and 1.26-fold at 24, 72, 120 and 144 hpi compared with the dsGFP injection group, respectively (Fig. 11E).

### 3.11. The Mortality Rate of dsRNA-injected L. vannamei after WSSV or V. alginolyticus Infection

**Figure 12:**
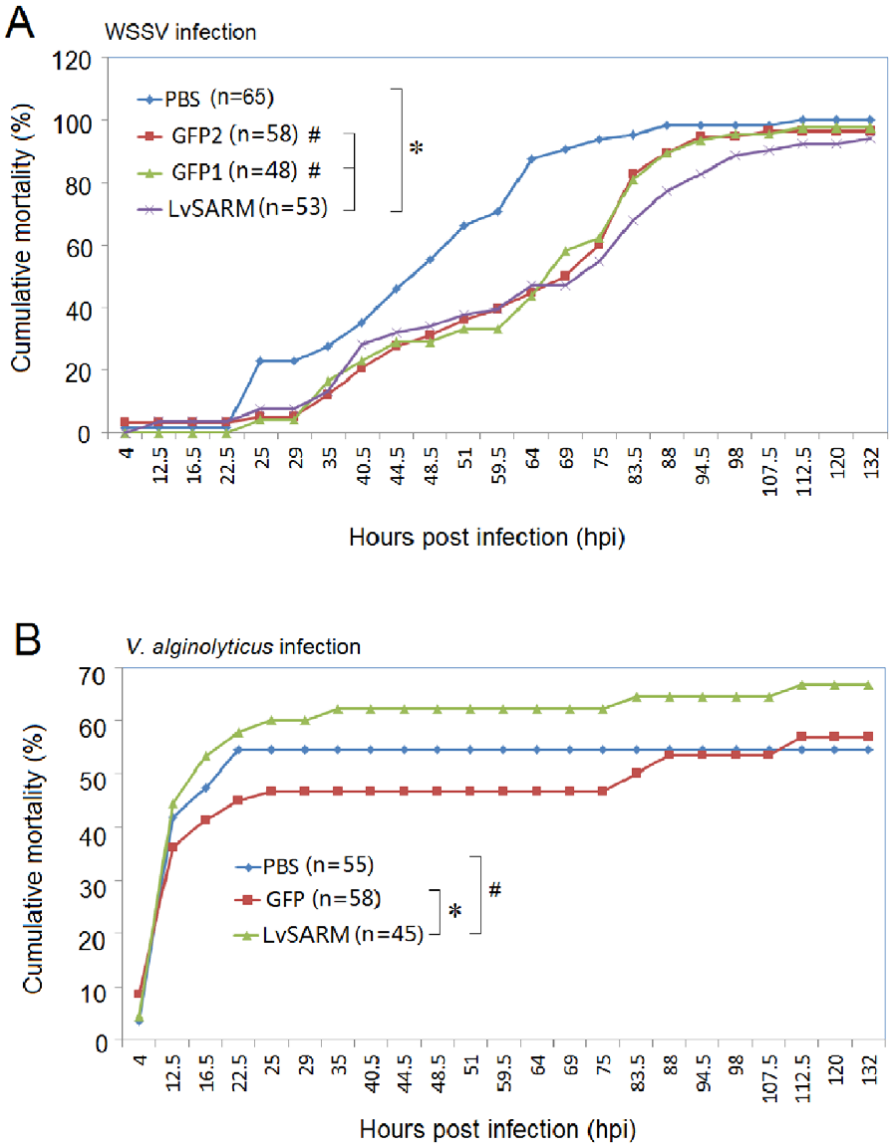
Silencing of *LvSARM* increases shrimp mortality after infection with *V. alginolyticus* but not WSSV. The cumulative mortalities of shrimps injected with PBS, dsGFP (control) or dsLvSARM after WSSV (A) or *V. alginolyticus* (B) infection were recorded. The chi-square statistic was calculated to assess the differences in mortality rates by comparing the mortality of dsLvSARM injection group with that of the dsGFP injection group (*p<0.05; #p>0.05).

WSSV is one of the most common and most destructive pathogens in shrimp aquaculture, and shrimp mortality can reach 100% within 3–10 days after infection. To further evaluate the role of LvSARM in shrimp immune responses, we performed WSSV infection experiments in dsRNA-injected *L. vannamei*. At 48 h after dsRNA injection, *L. vannamei* were infected with WSSV, and we found that injection of dsGFP and dsLvSARM could delay the initial outbreak of WSSV (Fig. 12A). At 144 hpi with WSSV, the PBS and dsGFP injection groups reached 100% mortality, but the dsLvSARM-injected group showed 94% mortality (Fig. 12A). Compared with the dsGFP injection group, dsLvSARM injection showed a slight protection from WSSV infection from 72 hpi to 144 hpi (Fig. 12A). *Vibrio* spp. is another important shrimp pathogen in China and Southern Asia. At 48 h after dsRNA injection, *L. vannamei* were infected with *V. alginolyticus*. We found that injection of dsGFP could protect shrimp from *V. alginolyticus* infection, while the dsLvSARM-injected shrimp showed higher mortality rate than the dsGFP- (p<0.05) or PBS-injected group (Fig. 12B).

## Discussion

In the course of combating invading microbes, NF-κB requires timely downregulation to avoid detrimental overactivation [16], [47]. Without regulation, overactivation of immune responses may cause chronic inflammatory and autoimmune diseases [16]. SARM and the inhibitor of NF-κB (IκB, called Cactus in *Drosophila*) are the most important negative regulators of the TLR-mediated NF-κB pathway, which is highly conserved from insects to humans [48], [49]. Cactus has been characterized as a repressor of the *Drosophila* Toll pathway and has been shown to bind to the NF-κB family protein Dorsal to block Dorsal’s nuclear localization signal [5], [6]. In *C. elegans*, SARM appears to have a positive Tol-1-independent function in immune responses [18], [19]. In contrast, whether insect SARM participates in immune responses is still unknown [18].

In mammals, SARMs only play a role in the TRIF-dependent signaling pathway, where they interact with TRIF, but not in the MyD88-dependent signaling pathway [15]. In contrast, invertebrate SARM, such as amphioxus SARM, can also interact with MyD88 and TRAF6 and inhibit the MyD88-dependent signaling pathway [50]. One recent study indicates that mammalian SARM can inhibit MyD88-mediated AP-1 activation through the MAPK pathway [51]. In *Drosophila* S2 cells, overexpression of LvSARM could inhibit the promoter activities of NF-κB-controlled shrimp AMPs, suggesting that LvSARM may function as a repressor in the Toll pathway (Fig. 8). LvSARM and LvTRAF6 could associate with each other, similar to amphioxus SARM and TRAF6 (Fig. 9). The cytoplasmic localization of LvSARM, which is similar to that of the mammalian SARM and amphioxus SARM, suggests that LvSARM may have similar functions in TLR-mediated NF-κB pathway [50], [51].

Like *C. rotundicauda SARM* [52] and amphioxus *SARM* [50], *LvSARM* was highly expressed in the muscle, which might suggest multi-functions of SARM such as development or cell death [53], [54] in addition to innate immunity. After WSSV infection, *LvSARM* expressed was decreased at 3 and 6 hpi comparing with the PBS injection group in the muscle. But at 24, 36 and 48 hpi, *LvSARM* was significantly upregulated (Fig. 6A). Meanwhile, we also found that, in the muscle, shrimp AMPs, including *LvPEN2*, *LvPEN3*, *LvPEN4* and *LvALF2*, were significantly downregulated at 24, 36 and 48 hpi (Fig. 6B, C, D, E). In the hemocyte, when *LvSARM* was upregulated at 36, 48 and 72 hpi, *LvPEN2*, *LvPEN3* and *LvPEN4* were downregulated (Fig. 6B, C, D). So there may be a negative correlation in mRNA expression between *LvSARM* and shrimp AMPs in the hemocyte and muscle after WSSV infection. Interestingly, we also found that, when *LvSARM* was upregulated at 24, 36, 48 and 72 hpi, viral *VP28* and *immediate-early gene 1* (*IE1*) were also upregulated in the hemocyte and muscle (Fig. S3). But whether this correlation between *LvSARM* overexpression and expression of viral genes is used by WSSV to avoid or manipulate shrimp immune responses remains to be determined.

Our dsRNA-mediated RNAi experiments showed that silencing of *LvSARM* could significantly increase the expression levels of *PENs* and *ALFs*, indicating that LvSARM may be a negative regulator in the shrimp Toll-NF-κB mediated pathway (Fig. 11). Given that the TRIF-dependent signaling pathway does not exist in invertebrates, it is likely that LvSARM participates in the Toll pathway via MyD88-dependent signaling, which is evolutionarily conserved from insects to humans [3], [5], [6]. This study also demonstrated for the first time that some shrimp AMPs, such as PENs and ALFs, may be regulated by a Toll signaling pathway similar to the regulation mechanism of *Drosophila* AMP genes by the Toll pathway. Although the expression levels of *PENs* and *ALFs* in *LvSARM*-silenced shrimps were upregulated under most conditions, the *LvSARM*-silenced shrimps were more susceptible to infection by *V. alginolyticus* than by WSSV (Fig. 12), suggesting that LvSARM might be indispensable in shrimp anti-*V. alginolyticus* responses.

**Figure 13:**
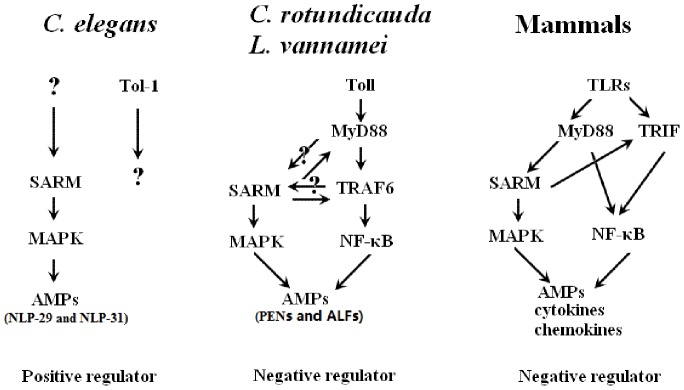
A proposed model for SARMs in the innate immune signaling pathways. SARM is a positive regulator of the MAPK signaling pathway in *C. elegans* for AMP regulation but functions as a negative regulator in the Toll pathway of *L. vannamei* and the TLR pathway of mammals.

Based on previous reports and this current study, we propose that the involvement of SARM in the MAPK pathway is conserved from *C. elegans* to mammals but has opposing roles in *C. elegans* and mammals (Fig. 13) [11], [18], [50], [51]. In *C. elegans*, SARM is a positive regulator of the MAPK pathway, while in mammals, SARM is a negative regulator of the MyD88-mediated MAPK pathway [10], [18], [51]. The involvement of SARM in the MyD88-dependent signaling pathway may have originated from an arthropod such as *C. rotundicauda* or shrimp (Fig. 13) [17]. In mammals, SARM retains its function in the MyD88-dependent MAPK pathway, but also acts as a suppressor, similar to arthropod SARMs [51]. However, this suppressive function of mammalian SARM is mediated via TRIF-dependent signaling leading to NF-κB activation, rather than by MyD88-dependent signaling [15]. Investigations regarding whether LvSARM participates in the shrimp MAPK pathway and of the interaction between LvSARM and shrimp MyD88 would be helpful to achieve a better understanding of the roles of SARM in the innate immune signaling pathways.

In the current study, full-length cDNA cloning, followed by cellular localization, expressions, and functional analyses of LvSARM, were performed. LvSARM is evolutionarily conserved and responds to microbial infections. Using a luciferase reporter assay and dsRNA-mediated RNAi, we demonstrated that LvSARM is a potential negative player of the shrimp Toll pathway in regulating the expression of *PENs* and *ALFs*. This study also extends our understanding of the evolution of the TLR signaling pathway.

## Supporting Information

Figure S1 **Multiple sequence alignments of the conserved regions of CeSARM, LvSARM, DpSARM, CrSARM, AmSARM, DmSARM, DrSARM, CgSARM, XtSARM and HsSARM. Two ARM domains, two SAM domains and the TIR domain of SARMs are boxed.**
(TIF)

Figure S2 **Schematic representations of the domain topology of CeSARM, LvSARM, CrSARM, DmSARM and HsSARM.**
(TIF)

Figure S3 **Expression of** ***VP28*** **and** ***IE1*** **in the hemocyte (A–B) and muscle (C–D) after WSSV infection.** The expression of viral *VP28* and *immediate-early gene 1* (*IE1*) began to decrease in the hemocyte but not in the muscle at 72 hpi. This may be due to apoptosis of shrimp hemocyte infected by WSSV (unpublished data).
(TIF)

## References

[1] Hoffmann JA, Kafatos FC, Janeway CA, Ezekowitz RA (1999) Phylogenetic perspectives in innate immunity. Science 284: 1313–1318. 10334979 10.1126/science.284.5418.1313

[2] Medzhitov R (2001) Toll-like receptors and innate immunity. Nat Rev Immunol 1: 135–145. 11905821 10.1038/35100529

[3] Akira S, Uematsu S, Takeuchi O (2006) Pathogen recognition and innate immunity. Cell 124: 783–801. 16497588 10.1016/j.cell.2006.02.015

[4] Takeuchi O, Akira S (2010) Pattern recognition receptors and inflammation. Cell 140: 805–820. 20303872 10.1016/j.cell.2010.01.022

[5] Valanne S, Wang JH, Ramet M (2011) The *Drosophila* Toll signaling pathway. J Immunol 186: 649–656. 21209287 10.4049/jimmunol.1002302

[6] Lemaitre B, Hoffmann J (2007) The host defense of *Drosophila melanogaster* . Annu Rev Immunol 25: 697–743. 17201680 10.1146/annurev.immunol.25.022106.141615

[7] Ramirez JL, Dimopoulos G (2010) The Toll immune signaling pathway control conserved anti-dengue defenses across diverse *Ae. aegypti* strains and against multiple dengue virus serotypes. Dev Comp Immunol 34: 625–629. 20079370 10.1016/j.dci.2010.01.006 PMC2917001

[8] Sabin LR, Hanna SL, Cherry S (2010) Innate antiviral immunity in *Drosophila* . Curr Opin Immunol 22: 4–9. 20137906 10.1016/j.coi.2010.01.007 PMC2831143

[9] Kemp C, Imler JL (2009) Antiviral immunity in *Drosophila* . Curr Opin Immunol 21: 3–9. 19223163 10.1016/j.coi.2009.01.007 PMC2709802

[10] Irazoqui JE, Urbach JM, Ausubel FM (2010) Evolution of host innate defence: insights from *Caenorhabditis elegans* and primitive invertebrates. Nat Rev Immunol 10: 47–58. 20029447 10.1038/nri2689 PMC2965059

[11] Leulier F, Lemaitre B (2008) Toll-like receptors-taking an evolutionary approach. Nat Rev Genet 9: 165–178. 18227810 10.1038/nrg2303

[12] Pujol N, Link EM, Liu LX, Kurz CL, Alloing G, et al (2001) A reverse genetic analysis of components of the Toll signaling pathway in *Caenorhabditis elegans* . Curr Biol : CB 11: 809–821. 11516642 10.1016/s0960-9822(01)00241-x

[13] O’Neill LA, Bowie AG (2007) The family of five: TIR-domain-containing adaptors in Toll-like receptor signalling. Nat Rev Immunol 7: 353–364. 17457343 10.1038/nri2079

[14] Zhang Q, Zmasek CM, Cai X, Godzik A (2011) TIR domain-containing adaptor SARM is a late addition to the ongoing microbe-host dialog. Dev Comp Immunol 35: 461–468. 21110998 10.1016/j.dci.2010.11.013 PMC3085110

[15] Carty M, Goodbody R, Schroder M, Stack J, Moynagh PN, et al (2006) The human adaptor SARM negatively regulates adaptor protein TRIF-dependent Toll-like receptor signaling. Nat Immunol 7: 1074–1081. 16964262 10.1038/ni1382

[16] Liew FY, Xu D, Brint EK, O’Neill LA (2005) Negative regulation of toll-like receptor-mediated immune responses. Nat Rev Immunol 5: 446–458. 15928677 10.1038/nri1630

[17] Belinda LW, Wei WX, Hanh BT, Lei LX, Bow H, et al (2008) SARM: a novel Toll-like receptor adaptor, is functionally conserved from arthropod to human. Mol Immunol 45: 1732–1742. 17980913 10.1016/j.molimm.2007.09.030

[18] Couillault C, Pujol N, Reboul J, Sabatier L, Guichou JF, et al (2004) TLR-independent control of innate immunity in *Caenorhabditis elegans* by the TIR domain adaptor protein TIR-1, an ortholog of human SARM. Nat Immunol 5: 488–494. 15048112 10.1038/ni1060

[19] Liberati NT, Fitzgerald KA, Kim DH, Feinbaum R, Golenbock DT, et al (2004) Requirement for a conserved Toll/interleukin-1 resistance domain protein in the *Caenorhabditis elegans* immune response. Proc Natl Acad Sci U S A 101: 6593–6598. 15123841 10.1073/pnas.0308625101 PMC404090

[20] Tauszig-Delamasure S, Bilak H, Capovilla M, Hoffmann JA, Imler JL (2002) *Drosophila* MyD88 is required for the response to fungal and Gram-positive bacterial infections. Nat Immunol 3: 91–97. 11743586 10.1038/ni747

[21] Leu JH, Yang F, Zhang X, Xu X, Kou GH, et al (2009) Whispovirus. Curr Top Microbiol Immunol 328: 197–227. 19216439 10.1007/978-3-540-68618-7_6

[22] Bachere E, Gueguen Y, Gonzalez M, de Lorgeril J, Garnier J, et al (2004) Insights into the anti-microbial defense of marine invertebrates: the penaeid shrimps and the oyster *Crassostrea gigas* . Immunol Rev 198: 149–168. 15199961 10.1111/j.0105-2896.2004.00115.x

[23] Bachere E (2000) Shrimp immunity and disease control-Introduction. Aquaculture 191: 3–11.

[24] Wang PH, Gu ZH, Huang XD, Liu BD, Deng XX, et al (2009) An immune deficiency homolog from the white shrimp, *Litopenaeus vannamei*, activates antimicrobial peptide genes. Mol Immunol 46: 1897–1904. 19232438 10.1016/j.molimm.2009.01.005

[25] Destoumieux D, Munoz M, Bulet P, Bachere E (2000) Penaeidins, a family of antimicrobial peptides from penaeid shrimp (Crustacea, Decapoda). Cell Mol Life Sci 57: 1260–1271. 11028917 10.1007/PL00000764 PMC11146768

[26] Woramongkolchai N, Supungul P, Tassanakajon A (2011) The possible role of penaeidin5 from the black tiger shrimp, *Penaeus monodon*, in protection against viral infection. Dev Comp Immunol 35: 530–536. 21199664 10.1016/j.dci.2010.12.016

[27] Li CY, Yan HY, Song YL (2010) Tiger shrimp (*Penaeus monodon*) penaeidin possesses cytokine features to promote integrin-mediated granulocyte and semi-granulocyte adhesion. Fish Shellfish Immunol 28: 1–9. 19748589 10.1016/j.fsi.2009.09.003

[28] Li CY, Song YL (2010) Proline-rich domain of penaeidin molecule exhibits autocrine feature by attracting penaeidin-positive granulocytes toward the wound-induced inflammatory site. Fish Shellfish Immunol 29: 1044–1052. 20816808 10.1016/j.fsi.2010.08.020

[29] Cuthbertson BJ, Deterding LJ, Williams JG, Tomer KB, Etienne K, et al (2008) Diversity in penaeidin antimicrobial peptide form and function. Dev Comp Immunol 32: 167–181. 17716729 10.1016/j.dci.2007.06.009 PMC2245800

[30] Somboonwiwat K, Marcos M, Tassanakajon A, Klinbunga S, Aumelas A, et al (2005) Recombinant expression and anti-microbial activity of anti-lipopolysaccharide factor (ALF) from the black tiger shrimp *Penaeus monodon* . Dev Comp Immunol 29: 841–851. 15978281 10.1016/j.dci.2005.02.004

[31] de la Vega E, O’Leary NA, Shockey JE, Robalino J, Payne C, et al (2008) Anti-lipopolysaccharide factor in *Litopenaeus vannamei* (LvALF): a broad spectrum antimicrobial peptide essential for shrimp immunity against bacterial and fungal infection. Mol Immunol 45: 1916–1925. 18078996 10.1016/j.molimm.2007.10.039

[32] Tharntada S, Ponprateep S, Somboonwiwat K, Liu H, Soderhall I, et al (2009) Role of anti-lipopolysaccharide factor from the black tiger shrimp, *Penaeus monodon*, in protection from white spot syndrome virus infection. J Gen Virol 90: 1491–1498. 19264668 10.1099/vir.0.009621-0

[33] Kadowaki T, Inagawa H, Kohchi C, Nishizawa T, Takahashi Y, et al (2011) Anti-lipopolysaccharide factor evokes indirect killing of virulent bacteria in kuruma prawn. In Vivo 25: 741–744. 21753127

[34] Han-Ching Wang K, Tseng CW, Lin HY, Chen IT, Chen YH, et al (2010) RNAi knock-down of the *Litopenaeus vannamei* Toll gene (LvToll) significantly increases mortality and reduces bacterial clearance after challenge with *Vibrio harveyi* . Dev Comp Immunol 34: 49–58. 19698743 10.1016/j.dci.2009.08.003

[35] de Lorgeril J, Gueguen Y, Goarant C, Goyard E, Mugnier C, et al (2008) A relationship between antimicrobial peptide gene expression and capacity of a selected shrimp line to survive a Vibrio infection. Mol Immunol 45: 3438–3445. 18486974 10.1016/j.molimm.2008.04.002

[36] Wang PH, Liang JP, Gu ZH, Wan DH, Weng SP, et al (2012) Molecular cloning, characterization and expression analysis of two novel Tolls (LvToll2 and LvToll3) and three putative Spatzle-like Toll ligands (LvSpz1–3) from *Litopenaeus vannamei* . Dev Comp Immunol 36: 359–371. 21827783 10.1016/j.dci.2011.07.007

[37] Wang PH, Wan DH, Pang LR, Gu ZH, Qiu W, et al (2012) Molecular cloning, characterization and expression analysis of the tumor necrosis factor (TNF) superfamily gene, TNF receptor superfamily gene and lipopolysaccharide-induced TNF-alpha factor (LITAF) gene from *Litopenaeus vannamei* . Dev Comp Immunol 36: 39–50. 21736897 10.1016/j.dci.2011.06.002

[38] Huang XD, Yin ZX, Jia XT, Liang JP, Ai HS, et al (2010) Identification and functional study of a shrimp Dorsal homologue. Dev Comp Immunol 34: 107–113. 19723535 10.1016/j.dci.2009.08.009

[39] Wang PH, Gu ZH, Wan DH, Zhang MY, Weng SP, et al (2011) The shrimp NF-κB pathway is activated by white spot syndrome virus (WSSV) 449 to facilitate the expression of *WSSV069* (*ie1*), *WSSV303* and *WSSV371* . PLoS One 6: e24773. 21931849 10.1371/journal.pone.0024773 PMC3171479

[40] Labreuche Y, O’Leary NA, de la Vega E, Veloso A, Gross PS, et al (2009) Lack of evidence for *Litopenaeus vannamei* Toll receptor (lToll) involvement in activation of sequence-independent antiviral immunity in shrimp. Dev Comp Immunol 33: 806–810. 19428481 10.1016/j.dci.2009.02.005

[41] Wang PH, Gu ZH, Huang XD, Liu BD, Deng XX, et al (2009) An immune deficiency homolog from the white shrimp, *Litopenaeus vannamei*, activates antimicrobial peptide genes. Mol Immunol 46: 1897–1904. 19232438 10.1016/j.molimm.2009.01.005

[42] Wang PH, Wan DH, Gu ZH, Deng XX, Weng SP, et al (2011) *Litopenaeus vannamei* tumor necrosis factor receptor-associated factor 6 (TRAF6) responds to Vibrio alginolyticus and white spot syndrome virus (WSSV) infection and activates antimicrobial peptide genes. Dev Comp Immunol 35: 105–114. 20816892 10.1016/j.dci.2010.08.013

[43] Pfaffl MW (2001) A new mathematical model for relative quantification in real-time RT-PCR. Nucleic Acids Res 29: e45. 11328886 10.1093/nar/29.9.e45 PMC55695

[44] Ho SH, Song YL (2009) Cloning of penaeidin gene promoter in tiger shrimp (*Penaeus monodon*). Fish Shellfish Immunol 27: 73–77. 19439182 10.1016/j.fsi.2009.05.001

[45] Li F, Wang D, Li S, Yan H, Zhang J, et al (2010) A Dorsal homolog (FcDorsal) in the Chinese shrimp *Fenneropenaeus chinensis* is responsive to both bacteria and WSSV challenge. Dev Comp Immunol 34: 874–883. 20363249 10.1016/j.dci.2010.03.008

[46] O’Leary NA, Gross PS (2006) Genomic structure and transcriptional regulation of the penaeidin gene family from *Litopenaeus vannamei* . Gene 371: 75–83. 16488092 10.1016/j.gene.2005.11.028

[47] Ragab A, Buechling T, Gesellchen V, Spirohn K, Boettcher AL, et al (2011) Drosophila Ras/MAPK signalling regulates innate immune responses in immune and intestinal stem cells. EMBO J 30: 1123–1136. 21297578 10.1038/emboj.2011.4 PMC3061042

[48] Hayden MS, Ghosh S (2008) Shared principles in NF-kappaB signaling. Cell 132: 344–362. 18267068 10.1016/j.cell.2008.01.020

[49] Liew FY, Xu D, Brint EK, O’Neill LA (2005) Negative regulation of toll-like receptor-mediated immune responses. Nat Rev Immunol 5: 446–458. 15928677 10.1038/nri1630

[50] Yuan S, Wu K, Yang M, Xu L, Huang L, et al (2010) Amphioxus SARM involved in neural development may function as a suppressor of TLR signaling. J Immunol 184: 6874–6881. 20483721 10.4049/jimmunol.0903675

[51] Peng J, Yuan Q, Lin B, Panneerselvam P, Wang X, et al (2010) SARM inhibits both TRIF- and MyD88-mediated AP-1 activation. Eur J Immunol 40: 1738–1747. 20306472 10.1002/eji.200940034

[52] Belinda LW, Wei WX, Hanh BT, Lei LX, Bow H, et al (2008) SARM: a novel Toll-like receptor adaptor, is functionally conserved from arthropod to human. Mol Immunol 45: 1732–1742. 17980913 10.1016/j.molimm.2007.09.030

[53] Osterloh JM, Yang J, Rooney TM, Fox AN, Adalbert R, et al (2012) dSarm/Sarm1 Is Required for Activation of an Injury-Induced Axon Death Pathway. Science 337: 481–484. 22678360 10.1126/science.1223899 PMC5225956

[54] Chen CY, Lin CW, Chang CY, Jiang ST, Hsueh YP (2011) Sarm1, a negative regulator of innate immunity, interacts with syndecan-2 and regulates neuronal morphology. J Cell Biol 193: 769–784. 21555464 10.1083/jcb.201008050 PMC3166868

