# Supplementary figures and images for: Litopenaeus vannamei Sterile-Alpha and Armadillo Motif Containing Protein (LvSARM) Is Involved in Regulation of Penaeidins and antilipopolysaccharide factors
Source: PLoS One. 2013 Feb 6;8(2):e52088. doi: 10.1371/journal.pone.0052088 (PMC3566147; doi:10.1371/journal.pone.0052088)

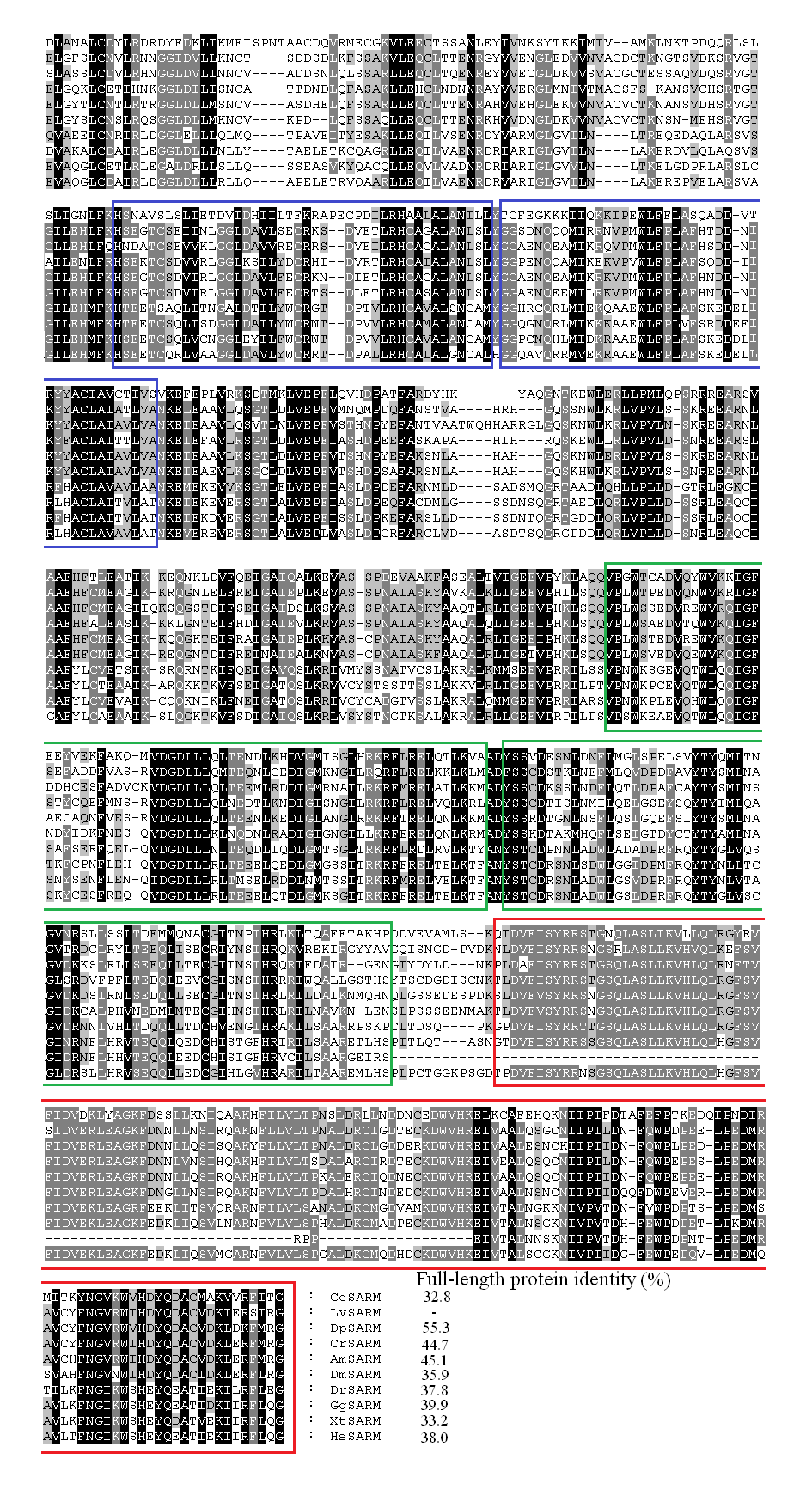

Supplement: Figure S1 — Multiple sequence alignments of the conserved regions of CeSARM, LvSARM, DpSARM, CrSARM, AmSARM, DmSARM, DrSARM, CgSARM, XtSARM and HsSARM. Two ARM domains, two SAM domains and the TIR domain of SARMs are boxed. (TIF) [file pone.0052088.s001.tif]

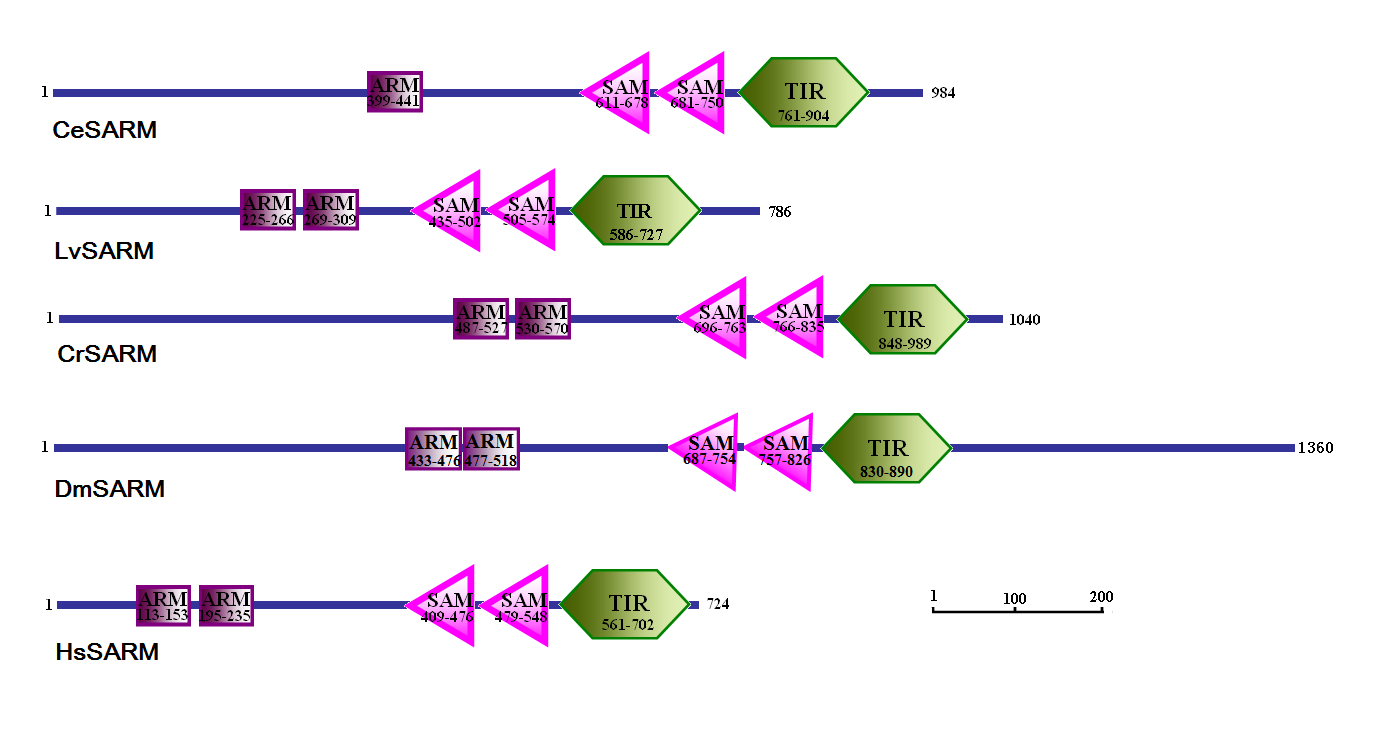

Supplement: Figure S2 — Schematic representations of the domain topology of CeSARM, LvSARM, CrSARM, DmSARM and HsSARM. (TIF) [file pone.0052088.s002.tif]

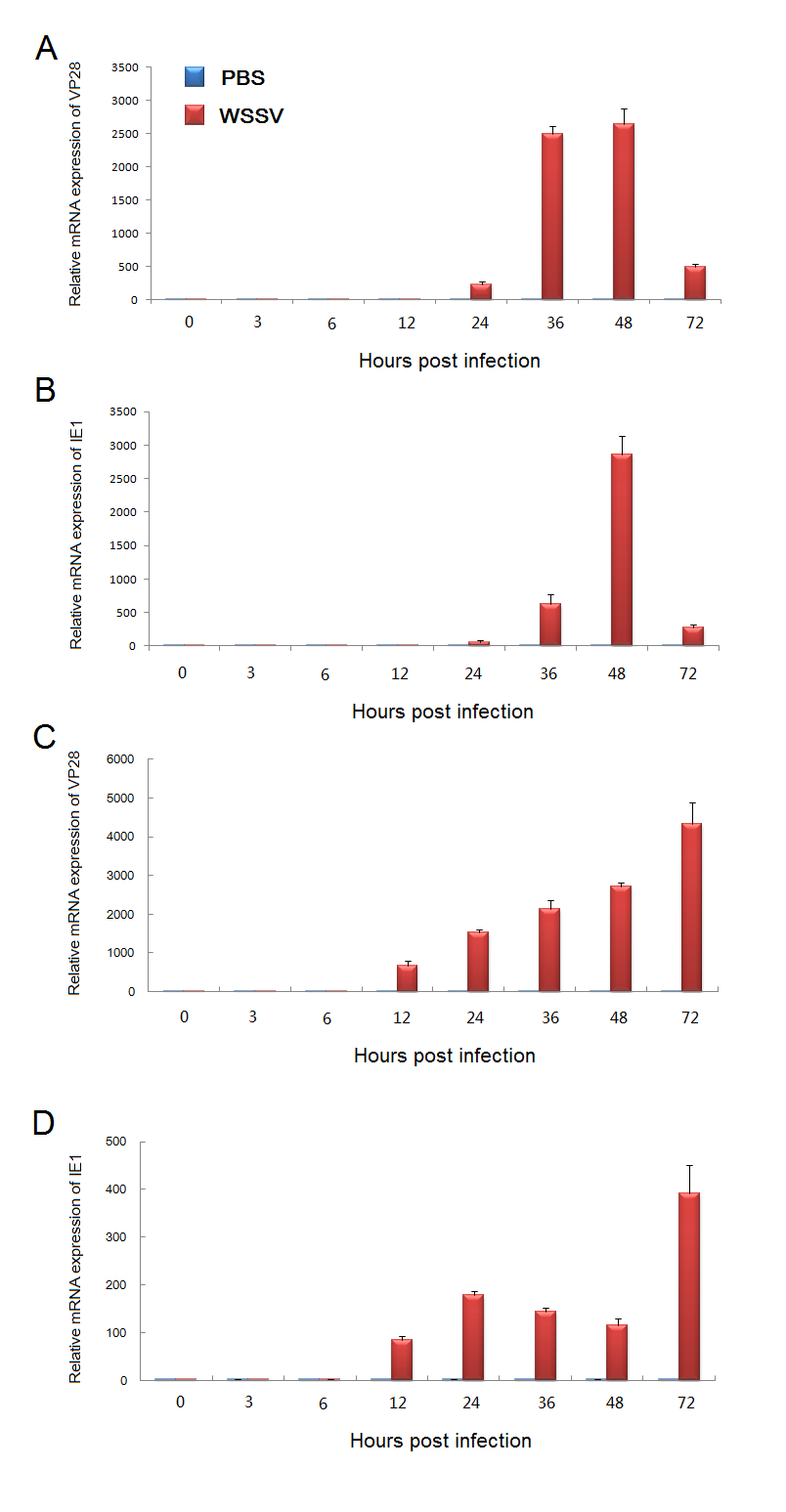

Supplement: Figure S3 — Expression of VP28 and IE1 in the hemocyte (A–B) and muscle (C–D) after WSSV infection. The expression of viral VP28 and immediate-early gene 1 (IE1) began to decrease in the hemocyte but not in the muscle at 72 hpi. This may be due to apoptosis of shrimp hemocyte infected by WSSV (unpublished data). (TIF) [file pone.0052088.s003.tif]
